# Monthly Summary

**Published:** 1877-01

**Authors:** 


### MONTHLY SUMMARY.

---

*Cure for Tetanus by Mechanical Measures.*—A patient, who was convalescent after various attacks of hemorrhage from the skin and mucous membranes, was wounded in the sole of the right foot and had partial tetanus result. There was considerable rigidity of the muscles of the cervical region and those presiding over the movements of the jaw. Sulphate of quinia and charcoal were followed by temporary relief only. Then C. thinking that the difficulty of injecting sufficient nutriment was due to the localization of the tetanic affections in the muscles named above, concluded to overcome the rigidity by forced movements of flexion; extension and rotation. This was continued till voluntary movement became possible. The jaws were then separated little by little from day to day, until they could be more and more widely opened, and the patient could himself take solid food. This treatment continued during the month of August, and by autumn the cure was completed.—*Chicago Med. Jour. and Ex.*

*Is Alcohol a Food*—In a previous number of *The Record*, Dr. Mays seems to desire to support the theory that alcohol is a food. The question presents itself, what is a food? that which supports life, or that which replenishes tissue?

Does alcohol support life? Does it contain those elements which replenish the tissue of the body, as they are day by day worn out and cast off?

Organic chemistry teaches that all tissue material supplies contain nitrogen as an essential part; that all force-giving foods are hydrocarbons. Alcohol contains no nitrogen; hence it cannot be a tissue food. Is it then a force-supplying food? No.

In the researches of Dr. Anstie, assisted by Dr. Dupre, the results of his experiments were unmistakable in their bearing on the question.

Hydrocarbons produce force by oxidation. Alcohol is a hydrocarbon, and, if consumed in the body, must produce heat, in order to rank among the heat-forming foods. Does alcohol cause an increase of animal heat? The experiments of Dr. Benjamin W. Richardson, of Edinburgh, on this question, go to prove that, instead of producing animal heat it diminishes the heat in the body. He divides the progressive changes of animal function from alcohol into four stages:

1st. Stage of excitement with relaxations and injection of the blood-vessels of the minute circulation with which we have become conversant.

2d. Stage of excitement, with muscular inability and deficient automatic control.

3d. Stage of rambling, incoherent, emotional excitement with loss of voluntary muscular power, and ending in helpless un consciousness.

4th, and final stage, where the heart begins to fail, and in which death, in extreme instances of intoxication, closes the scene.

In the first stage the temperature may rise to one-half degree Fahr. in man. In the second stage the heat comes down to its natural standard, and then declines below what is natural. During the third state the fall in temperature rapidly increases, and, as the fourth stage is reached, it approaches a point that is actually dangerous. There is, also, during this stage profound coma, or sleep, and while this lasts, the temperature remains reduced. And here we have a diagnostic point between the sleep of apoplexy and the sleep of drunkenness, for in apoplexy the temperature remains normal.

These researches, although differing in results from Drs. Anstie and Dupre, would, I doubt not, have been reached by Dr. A., had not his useful life been cut short at the time it was, —*Med. Record*,

*Medical Women.*—The King and Queen's College of Physicians of Dublin has at length determined to admit a woman to its privileges of license. Although this fact startles some of our English brethren, we believe that the college has not taken a very dangerous departure. We see no reason why it should not, like medical institutions in this and other countries, share the responsibility of solving a vexatious question. It is useless to rail against the women studying medicine. They will do it in spite of male opposition, and have a right at least to try.

They believe they have a sphere in medicine, and we should rather help than hinder them in finding it. Whether they are specially fitted for medical work is not now the question, but whether we will give them an opportunity to work out their own salvation. If we persist in persecuting them, we must only wait the longer for the problem to be solved. The *Lancet* says: "We believe woman's work is to console and support man, not to usury his functions." This view is eminently sound and as a general rule is admitted by all. But all general rules have their exceptions, and it is with these exceptions that we mainly have to deal. It is useless for the *Lancet* to say that "unwomanly women alone can aspire to unwomanly work," for it has no effect. The women view the questfon from an altogether different stand point, and are willing to shoulder all the opprobrium of the new situation. It is entirely gratuitous for us to bewail their sacrifice of the tender qualities which we call lovable and womanly; if such be sacrificed, what real responsibility have we in the matter? The simple fact narrows itself down to this; some women believe that they are specially qualified to study medicine, and as a profession we believe to the contrary. Neither party can be satisfied until a fair trial is allowed. The right for a trial no one must deny, and the college in question does no more than grant it. The English profession will not be ruined by the course adopted, neither will any extraordinary number of women avail themselves of the advantage offered.

In New York we met the issue long ago, and would have settled the question if we were not forced to acknowledge that "one swallow does not make a summer." It is true that we have a very fine specimen of a swallow, but the summer is not yet.—*Med. Record.*

---

*How they Pull Children's Teeth in Paris.*—In the children's hospital in Paris, the nurse goes round at eight A. M. and gives to each child under sentence from thirty to fifty grains chloral hydrate. The dentist follows in one hour, and the child wakes up an hour or two afterwards and wonders what has become of its tooth.—*Pacific Med. and Surg. Journal.*

*Congenital Cleft Palate.*—Mr. Francis Mason has at the present time under observation, at St. Thomas's Hospital, several interesting cases of congenital cleft palate, which he is treating by the application of strong nitric acid alone, and consequently without the use of the knife. The ages of the patients vary from a few weeks to several years. Mr. Mason thinks that this method of effecting union is especially applicable to cases in which the cleft is of average extent, and even where the hard palate is partially implicated. In more severe instances the ordinary operation may be required.

Mr. Mason finds that the application of the acid is attended with no pain or inconvenience whatever to the patient, and although the cure is more slowly accomplished, it hos the advantage of being sure, and of completely closing the fissure in the most perfect manner, without the risk of the parts giving way, either wholly or partially, as too often happens after the usual operation of staphyloraphy, A further gain seems to be that the cases may be dealt with as out-patients, as in all the examples now under notice. Mr. Mason, after many trials, prefers the strong nitric acid to any other form of caustic. We shall continue to watch the progress of these cases, and give the results on a future occasion.—*London Lancet.*

---

*Chloral Plaster in Neuralgia.*—Dr. Solari, of Marseilles, says the *Medical Examiner*, recommends the chloral plaster as an excellent application in cases of neuralgia and of nervous pains resulting from exposure to cold. The plaster is easily prepared by powdering the chloral over a common pitch plaster, one to twe scruples of chloral for every four square inches of the plaster. Care is taken not to incorporate the chloral with the pitch. It is applied for twenty-four to forty-eight hours; when removed, the skin is found covered by a number of small vesicles; these are opened, and the part then covered with a cerate dressing. Generally speaking, it will be found that the pain has disappeared before the vesicles heal. Dr. Solari states that numerous cases of lumbago, intercostal and other forms of neuralgia, etc., have been rapidly cured by this simple method.—*American Medical Weekly.*

---

*Woman in Medicine.*—One of the lady students educated at the London School of Medicine for Women passed the examination in anatomy and physiology at Paris last week. The marks given at this examination are in four grades, and the highest were obtained by the lady in question, the examiners remarking that the knowledge of the subject she had shown was a proof not only of her own industry, but of the sound practical instruction she had received.—*Med. Press and Circular.*

*Hydrophobia Cured by Inhalation of Oxygen.*—We have the authority of Schmidt and Lebedew for the following case:—

A girl, aged twelve, was bitten by a rabid pup, in the hand, on January 7th, 1876. The wound extended into the subcutaneous cellular tissue: there was scarcely any bleeding; it was at once cauterized with lapis, and had entirely healed on the seventh day. The child had suffered an attack of diphtheria three months before, which had left paralytic aphonia behind it. About the time when the wound closed she was observed to be uncommonly excitable. On the seventeeth day severe dyspnœa suddenly set in; inspiration free; expiration difficult, inturrupted in character; deglutition almost impossible; pulse rapid; fingers contracted. In the course of twenty four hours neither urine nor fæces were voided. The inhalation of about three cubic feet of oxygen produced immediate amelioration of the symptoms, and within two and a half hours apparantly restored her to her former condition of health. The next day she had a more severe attack, with tonic spasm of the muscles of the back and limbs; respiration spasmodic; complete loss of consciousness. These symptoms were relieved in forty-five minutes by the inhalation of oxygen. Slight remaining dyspnœa was treated in the same manner for the following ten days, with the addition of *camphora monobromato*, which was given for three weeks. In the first part of February she had paralysis of both lower extremities for two weeks; since then she has been entirely well, excepting the aphonia which existed before she was bitten.—*Wratschebnija Wedomosty.*

---

*Irritating Effects of Salicylic Acid.*—That salicylic acid is not so harmless a body as is generally supposed is shown by its action on the skin. In preparing 4 and 10 per cent. salicylic wodding, a momentary dip of the hand into the solution caused the skin to scale off for several days. The author has also observed that the acid hinders the action of pepsine on albumen.

A mixture of 2 parts albumen, 5 drops of hydrochloric acid, 30 parts distilled water, 0.3 pepsine, and 0.1 salicylic acid, had a putrid smell after about 8 days. Would it be equally inactive in the hot summer days if milk, flesh, wine, beer, etc., be treated with the acid for the purpose of preserving them? Cantharides and belladonna plaster could not, by painting over with a spirituous solution of salicylic acid, be preserved from moulding. The article is, therefore, not infallible, and we await further precise experiments to determine its exact value in such cases.—*Ibid.—Jour. Med. News.*

*On the Local Action of Atropia.*—A. Zeller, of Heidelberg, says: Several authors had observed already that a solution of atropia caused a contraction of vessels when applied to a frog's web-membrane. Hayden thought that this contraction was due to reflex action. Then other observers noticed that an injection of a certain quantity of atropia is followed by a dilatation of the smaller vessels.

The author intended to search for the conditions under which the changes in the calibre of the vessels take place. For this purpose he irrigated the tongue of the rana temporaria with solutions of the sulphate of atropia of different strength. The experimenter had found beforehand that a *watery* solution of the sulphate of atropia dissolves the coloring matter of the red blood corpuscles, *swells* the white blood corpuscles and makes them *transparent*, so that not only their nuclei might be seen, but numerous minute granules in them, being in a state of motion, whereas no signs of amœboid movement were observed.

To avoid the action of the watery solution of the atropia upon the blood corpuscles, which resembles very much the action of distilled water, the author dissolved the sulphate in an indifferent neutral solution of salts (chloride of sodium.) This solution inhibits only the white blood cells from the amœboid motion.

Only the arteries dilate more or less when irrigated with a solution of one half to one per cent. sulphate of atropia in water or in a three fourths per cent. solution of chloride of soda. At the same time, the blood flows more rapidly, which phenomenon is especially to be seen in the *capillaries* and *veins*. If wounds are irrigated with the latter solution, the emigration of white blood corpuscles is soon interrupted, the blood cells become globular and get granulated. Only when the irrigation has ceased for an hour or longer the emigration commences anew.—*Virchow's Archives.*

---

*Deaths from Chloroform.*—The *Ohio Medical Recorder* for October, 1876, reports a case of death from the inhalation of chloroform occurring in a young woman aged 30, to whom it waa administered for the extraction of a tooth.

Another case is reported in the *Pacific Med. and Surg. Journal* for the same month. The patient was a man who resided in Madison County, Indiana, and the anæsthetic was administercd for the purpose of extracting a thorn from his foot.

Dr. W. T. McNutt reports (*Pacific Med. and Surg. Journal*, July, 1875) a case of this which occurred during the reduction of dislocated shoulder joint.—*Med. Newt.*

*Carbolic Acid as a Local Anæsthetic.*—Dr. Bergonzini, in the *Presse Med. Belge*, Oct. 8, recommends a new painless mode of opening abscesses. It consists in placing in contact with the skin for three or five minutes a mixture formed of one part of glycerine and two parts of carbolic acid. It produces neither redness nor swelling, and may be employed with advantage in autoplastic operations, and in the treatment of superficial neuralgia.

The anæsthetic properties of carbolic acid were first pointed out more than four years ago by Dr. J. H. Bill, U. S. Army, in an interesting paper in the *Am. Jour. of the Medical Sciences* for July, 1872.

---

*To Remove Mother's Marks.*—Mr. Balmanno Squire gives the following directions for removing what are called "port wine marks."

"Freeze the part to be operated on, and thus render it perfectly insensible (by means of the ether spray apparatus), then scratch it with an ordinary cataract needle with parallel scratches, then place a piece of blotting paper on it before it has thawed, and consequently before it has begun to bleed, and press the blotting paper firmly on the scratched skin for five minutes.—*Med. and Surg. Reporter.*

---

*Mastic Chewing.*—We adverted, some weeks ago, to the American "chewing gum." In the Orient they use gum mastic, and a correspondent of the *Chemist and Druggist* says he has been assurred by some of the first dentists in Constantinople shat mastic chewers suffer comparatively little from toothache. and that their principal patients are among those who have not adopted the habit. Even the little children chew mastic, and a mother or sister will give her own special piece to a noisy young ten-year old to keep him or her quiet. It is very odd to a freshly-arrived European, on paying a morning visit to some Greek or Armenian beauty, to see her take a large "quid" of what appears to be dentist's modeling wax out of her handsome mouth and deposit it by her side on the divan, so that her flow cf language may not be interfered with.—*Mea. & Surg. Reporter.*

---

*A Sensible Precaution.*—When lunar caustic is used in the oral cavity, and toward the tonsils and larynx, fears may be entertained that the stick may break and cause dangerous symptoms. To make such an unpleasant accident impossible, Dr. Mettenkeimer place the caustic in a little bag of gauze, through the meshes of which the former acts completely, the escape of she stick being effectually prevented. Of course the gauze thould be changed at each cauterization, as the meshes are liable to get obstructed by moisture, and even to be destroyed by the caustic.—*Lancet.*

